# Profiling haplotype specific CpG and CpH methylation within a schizophrenia GWAS locus on chromosome 14 in schizophrenia and healthy subjects

**DOI:** 10.1038/s41598-020-61671-2

**Published:** 2020-03-13

**Authors:** Margarita Alfimova, Nikolay Kondratyev, Arkadiy Golov, Vera Golimbet

**Affiliations:** Mental Health Research Center, Department of Clinical Genetics, Moscow, Russian Federation

**Keywords:** Genetics, Molecular biology, Biomarkers, Diseases

## Abstract

Interrogating DNA methylation within schizophrenia risk loci holds promise to identify mechanisms by which genes influence the disease. Based on the hypothesis that allele specific methylation (ASM) of a single CpG, or perhaps CpH, might mediate or mark the effects of genetic variants on disease risk and phenotypes, we explored haplotype specific methylation levels of individual cytosines within a genomic region harbouring the *BAG5*, *APOPT1* and *KLC1* genes in peripheral blood of schizophrenia patients and healthy controls. Three DNA fragments located in promoter, intronic and intergenic areas were studied by single-molecule real-time bisulfite sequencing enabling the analysis of long reads of DNA with base-pair resolution and the determination of haplotypes directly from sequencing data. Among 1,012 cytosines studied, we did not find any site where methylation correlated with the disease or cognitive deficits after correction for multiple testing. At the same time, we determined the methylation profile associated with the schizophrenia risk haplotype within the *KLC1* fourth intron and confirmed ASM for cytosines located in the vicinity of rs67899457. These genetically associated DNA methylation variations may be related to the pathophysiological mechanism differentiating the risk and non-risk haplotypes and merit further investigation.

## Introduction

Schizophrenia is a common, highly heritable disorder characterized by positive, negative, and cognitive symptoms. Large genome-wide association studies (GWAS) of the Psychiatric Genomics Consortium (PGC) have identified more than 100 genomic regions that are significantly associated with schizophrenia^1,2^. The GWAS results aggregated into polygenic scores explain a significant, albeit insufficient for clinical practice, proportion of the heritability of the disorder and correlate with a wide range of other phenotypes predictive of schizophrenia onset and outcomes^3–6^. However, to unravel the pathogenesis of schizophrenia and to find markers for targeted therapy, it is necessary to understand the role of individual risk loci regarding both gene expression and clinical phenotypes.

Because most schizophrenia risk variants are non-coding, interrogating epigenetic and other regulatory processes within the risk loci holds promise for identifying mechanisms by which each locus influences the development of the disease. To date, many investigators have relied on array-based whole-methylome studies (MWAS). The MWASs have found changes in DNA methylation in the blood and brain of schizophrenia patients (reviewed in ref. ^7^), and current research focuses on the degree of colocalization between the index PGC GWAS loci and differentially methylated sites to find potential causal variants and target genes^8–11^. Such studies, along with advantages, have some limitations to delineate the biological significance of individual loci.

First, there is evidence that changes in methylation of a single CpG can affect transcription and serve as a more accurate predictor of gene expression or a behavioural phenotype than an average promoter/gene body methylation^12^. Though microarrays used in schizophrenia MWASs (mainly Illumina 450 k) provide comprehensive coverage of genes and CpG islands, they have not been specifically targeted to schizophrenia. For this reason, the microarrays are limited in the number of cytosines they can assess within the disease-associated loci. In addition, a sizeable proportion of the CpG sites tested is not relevant to the disease, which reduces the likelihood of detecting relevant sites^13^. Second, MWASs have provided very limited information on cytosine methylation in the non-CpG context (CpH). Meanwhile, CpH methylation presents at significant levels in human pluripotent stem cells, oocytes and neurons^14–22^. Moreover, the idea that CpH methylation is more frequent than originally thought and may have a functional role in many somatic cell types is gaining acceptance^23^. Third, haplotype-dependent allele-specific methylation (ASM) is proposed to mediate or mark the effects of genetic variants on gene expression and disease susceptibility, with genetic variants exerting an influence on DNA methylation *in cis*. Methylation quantitative trait loci (mQTL) are enriched in the schizophrenia risk loci^8,24,25^ and may explain 25% of the genetic variation of the disorder^26^. In addition, mQTL are often colocalized with expression QTL (eQTL), which is in line with the hypothesis that ASM may be a cause or a result of allele specific transcription factor binding^27,28^. Of particular interest is methylation associated with polymorphisms that create or abolish CpG sites (CpG-SNP) because CpG-SNPs account for the existence of most mQTL^29,30^. However, methylation at CpG-SNPs is poorly captured by conventional MWASs.

The foregoing suggests that interrogating haplotype-dependent allele-specific methylation at single-base resolution might help find markers of schizophrenia and provide insight into biological relevance of the statistically identified risk loci. Sequencing-based approaches that capture relatively short DNA fragments have been recently applied to explore the role of allele-specific methylation in schizophrenia^31,32^. Further progress in understanding the interplay of genetic and epigenetic factors in disease risk is expected to be achieved by a direct assessment of DNA modification in long reads enabling more robust genetic and epigenetic haplotypic assessment^33^.

The emerging long-read technologies, such as the third-generation single molecule real time (SMRT) sequencing from Pacific Biosciences (PacBio) and Oxford Nanopore sequencing, can sufficiently impact this area of research and overcome limitations inherent for array-based and short-read sequencing methods, as these technologies allow long-range characterization of methylation patterns and generation of phase information over long contiguous segments. Specifically, an innovative method combining bisulfite conversion with SMRT sequencing (SMRT-BS) has been proposed for targeted CpG methylation analyses. The method makes it possible to measure methylation of each cytosine within long amplicons (up to ~1.5–2.0 kb) and to determine haplotypes directly from sequencing data with high accuracy^34,35^.

The present study took advantage of SMRT-BS to examine haplotype dependent methylation states of CpGs and CpHs in one of the top schizophrenia risk loci (chr14:103,996,234–104,184,834, hg19/GRCh37)^1,2^ in peripheral blood of patients and controls. This is one of the index loci whose role in the disease pathophysiology is completely unclear, as the genes annotated to it (*APOPT1, BAG5, KLC1, TRMT61A, XRCC3, CKB, PPP1R13B, LC1, AL0498, ZFYVE*2*1*) are not conventional candidates for schizophrenia and are not relevant to major hypotheses of the aetiology and treatment of the disease^1^. Given that within each risk locus there may be multiple genetic variants impacting the disease risk to varying degrees, with some acting via a DNA methylation variability^33,36^, exploring methylation within this locus could provide epigenetic markers of schizophrenia and related phenotypes and shed light on mechanisms by which the locus is involved along the causal pathway to the disease. In particular, since this genomic region is associated with cognition in a large GWAS of intelligence (chr14:103,822,687–104,174,123)^37^, it is a promising target to search for epigenetic markers of cognitive deficits in schizophrenia patients.

**Figure 1:**
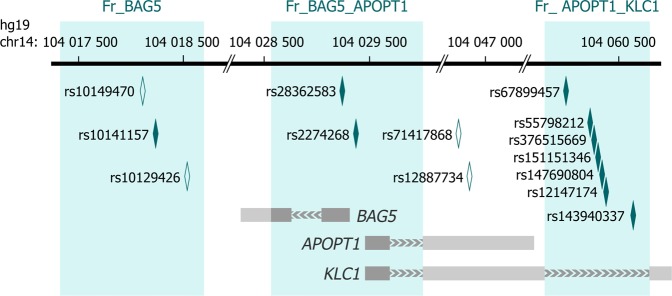
Schematic representation of the genome region. The scheme depicts three target fragments shaded in blue, simplified structures of genes in grey (light grey designates gene parts locating outside the target fragments, without differentiating exons and introns), polymorphisms directly determined from the bisulfite data (dark diamonds), and other important polymorphisms (uncoloured diamonds).

Previous studies of schizophrenia cases revealed differential methylation of several cytosines located within this locus, primarily in the *KLC1* body^8,10,38–40^. Here, we continued characterization of local methylation patterns with the aim to provide detailed information on the relationships between genetic variants and methylation status of single cytosines and to search for markers of schizophrenia and cognitive deficits. We studied three ~1.0 kb DNA fragments with different genetic features, namely with promoter, intron and intergenic locations (Fig. 1, see also Supplementary Table S1 for the fragments’ boundaries and numbers of cytosines). The fragments were selected from the region surrounding the PGC GWAS tag SNPs, rs12887734 (PGC2 GWAS) and rs71417868 (PGC2-CLOZUK), based on criteria described in ref. ^41^. Specifically, we focused on CpG-SNP rich DNA segments. The annotation of polymorphisms with MAF > 20%, located within the selected fragments, is given in Supplementary Table S2.

The region surrounding the tag SNPs harbours the *BAG5*, *APOPT1* (also known as *COA8*) and *KLC1* genes. BAG5 and APOPT1 have anti- and proapoptotic functions, respectively. BAG5, in addition, is involved in neurodevelopment and is considered in the context of the hypothesis of impaired angiogenesis in schizophrenia aetiology^42^. *KLC1* encodes one of the light chain isoforms of kinesin-1, a microtubule-based motor protein playing the important role in position and transportation of different biomolecules within a cell. Kinesin-1 is the most abundant of the motor family in the brain and is involved in neuronal differentiation^43^.

The first target fragment, hereinafter called Fr_BAG5_APOPT1, is a common promoter of *BAG5, APOPT1* and one of the *KLC1* isoforms, embedded in a CpG island. A local polymorphism, rs28362583, is in strong linkage disequilibrium (LD) with the schizophrenia index SNP rs12887734 (*D*′ = 0.99, *r*^2^ = 0.92, European populations)^44^ and with rs7148456 (*D*′ = 0.99, *r*^2^ = 0.98) affecting alternative splicing of *APOPT1* in the brain^45^. The second DNA fragment (Fr_APOPT1_KLC1), located within one of the *KLC1* introns, is an enhancer regulating transcription from the above-described promoter (GeneHancer ID: GH14J103593)^46^. It contains rs67899457, which is an mQTL in the blood^30^ and an eQTL for several genes in blood and brain tissues^47,48^. Given this, rs28362583- and rs67899457-driven ASM is a candidate mechanism by which this locus is associated with schizophrenia. The third fragment (Fr_BAG5) situates downstream of *BAG5* in an intergenic area. Recent large-scale GWASs have identified local SNPs as index variants for several psychiatric and cognitive phenotypes, such as autism (rs10149470)^49^, major depression (rs10149470)^50^, intelligence (rs10141157; rs10149470)^37,51^, and educational attainment (rs10129426)^52^. We therefore expected that ASM within this fragment might be related to cognitive alterations in psychiatric conditions and would correlate with cognitive deficits in schizophrenia.

Based on the hypothesis that ASM of a single CpG, or perhaps CpH, might mediate or mark the effects of genetic variants on disease risk and phenotypes, we determined haplotype specific methylation levels of each cytosine (CpG and CpH), residing within these three fragments, in the peripheral blood of schizophrenia patients and healthy controls and analysed their relationship to the disease and cognitive functioning.

## Results

### Sample characteristics

The patient and control groups were similar in age and sex composition but differed in education (χ2 = 5.13, *p* < 0.001) and cognitive scores (patients’ mean ± s.d., 38.66 ± 6.53 Т-scores; controls, 49.63 ± 5.04 Т-scores; *t* = 11.31, *p* < 0.001; see Methods and Supplementary Table S3 for a detailed description of the composite cognitive score). We included in the analysis only heterozygotes with at least 5× coverage per strand (see Methods). For this reason, the proportion of heterozygotes in the sample was reduced and the distribution of genotypes in many cases deviated from Hardy-Weinberg equilibrium. For the same reason, the fragments slightly differed in the number of people whose data were included in the analysis. However, for each fragment, cases and controls did not differ in age, gender, mean coverage, and frequencies of genotypes and haplotypes, and patients demonstrated significantly decreased cognitive scores compared to controls (the sample’s characteristics can be found in Supplementary Table S4).

### Fr_BAG5_APOPT1

**Figure 2:**
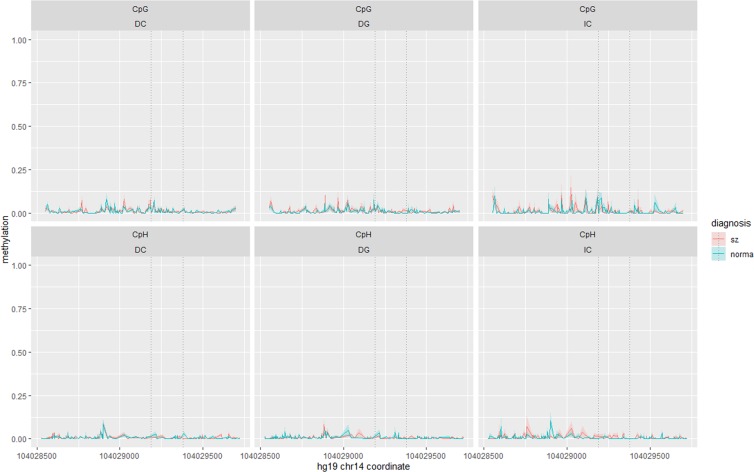
Allele specific methylation at CpG (top panel) and CpH (bottom panel) sites within Fr_BAG5_APOPT1 in schizophrenia patients (red line) and controls (green line). Average methylation with standard errors (coloured shaded areas) is shown separately for three possible haplotypes determined by rs28362583 and rs2274268 – DC, DG and IC, where ‘D’ designates deletion and ‘I’ designates insertion at rs28362583. Dotted vertical lines show positions of rs28362583 and rs2274268. IC is a schizophrenia risk haplotype.

Data of 49 patients and 50 controls were analysed. We determined methylation levels of 425 cytosines, including 145 CpGs, with the mean coverage of ~17. The whole region was practically unmethylated in accordance with its genomic features (promoter, CGI), with no influence of age and gender on methylation being detected. For both CpGs and CpHs, mean methylation levels (M) were less than 7% (Fig. 2).

We next discarded unmethylated cytosines (mean M ≤ 1%) and analysed the other 84 cytosines, including 49 CpGs. The fragment contains eight common polymorphisms. To choose haplotypes for analysis for this and the other fragments, we considered polymorphisms with MAF > 20%, took into account LD in European populations and the possibility to evaluate genotypes from bisulfite-converted DNA. As a result, within the Fr_BAG5_APOPT1 fragment we analysed the effect of haplotypes determined by two polymorphic sites − rs28362583 and rs2274268, which yield three common haplotypes in European populations. Rs28362583 is an indel mutation, the insertion of CCG creating an additional CpG. SNP rs2274268 is a missense C > G mutation in *KLC1*. The major allele C moderately correlates with the presence of the insertion at rs28362583 (*D*′ = 1.0, *r*^2^ = 0.20).

Average methylation values for each cytosine within the Fr_BAG5_APOPT1 fragment by group and haplotype and nominally significant *p*-values (*p* < 0.05) for every analysis conducted are given in Supplementary Table S5. No ASM was detected within the fragment. There was only one site, the CpG59_29024 (the cytosine’s name shows its serial number and the last five digits of the chromosome coordinate), where the difference between patients and controls was nominally significant in both the logistic regression adjusted for age, gender and haplotype and the matched pairs analyses controlling for haplotype and coverage. The cytosine was more frequently methylated in patients with the risk haplotype IC compared to controls with the same haplotype. However, the difference did not survive correction for multiple testing (false discovery rate, FDR < 0.05). Haplotypes and methylation did not predict cognitive scores in linear regression models adjusted for age, gender and diagnosis (nominally significant *p*-values can be found in Supplementary Table S5).

### Fr_APOPT1_KLC1

**Figure 3:**
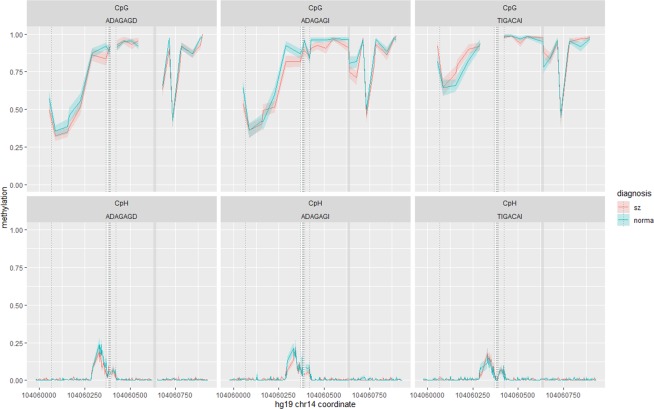
Allele specific methylation at CpG (top panel) and CpH (bottom panel) sites within Fr_APOPT1_KLC1 in schizophrenia patients (red line) and controls (green line). Average methylation with standard errors (coloured shaded areas) is shown separately for three possible haplotypes formed by polymorphisms rs67899457, rs55798212, rs376515669, rs151151346, rs147690804, rs12147174, and rs143940337 – ADAGAGD, ADAGAGI and TIGACAI, where ‘D’ designates deletion and ‘I’ designates insertion. Dotted vertical lines show positions of rs67899457, rs55798212, rs376515669, rs151151346, rs147690804, rs12147174, and rs72710744. The INDEL polymorphism rs143940337 is shown by the thick grey vertical line. TIGACAI is a schizophrenia risk haplotype.

Methylation levels of 259 cytosines, including 24 CpGs, with the mean coverage of ~16, were determined in 54 patients and 50 controls. The levels did not associate with age or gender. All CpGs were methylated, with 12 CpGs were intermediately methylated (20% < mean M < 80%) and 12 CpGs were hypermethylated (mean M > 80%). At the same time, the majority (84%) of CpHs were unmethylated (mean M < 1%) (Fig. 3).

Against this background, a short DNA segment with methylated CpHs stood out (chr14:104,060,285–104,060,422). This segment spanned 138 bp and included 33 CpHs, five СpGs and seven common polymorphisms, six of which being CpG-SNPs. Its average CpH methylation level was M = 8.5 ± 6.8% (vs. mean M = 0.4 ± 0.3% for the remaining CpHs within Fr_APOPT1_KLC1, *p* < 0.001). To quantify the specificity of this segment further, we compared it with the rest of the Fr_APOPT1_KLC1 fragment (815 bp, 202 CpHs, 19 CpGs, 4 common polymorphisms) on the proportions of cytosines, CpGs and SNPs and examined correlations between cytosines within this segment using an exploratory factor analysis. The segment did not differ from the rest of the fragment in the percentage of cytosines or CpGs, but the percentage of SNPs here was significantly higher (*p* < 0.001).

**Figure 4:**
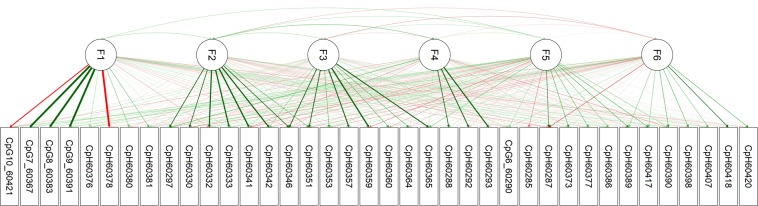
Path diagram showing results of an exploratory factor analysis of cytosines located within the Fr_APOPT1_KLC1 methylated segment (chr14:104,060,285–104,060,422). Data for the combined sample of patients and controls are presented. The last five digits of a cytosine’s coordinate are shown in the cytosine’s name. Green arrows designate positive loadings and red arrows designate negative loadings, with the line thickness corresponding to the strength of the connections. Of note, CpH60378 resides at the CpG-SNP rs376515669.

Results of the factor analysis are provided in Fig. 4 and Supplementary Tables S6a–d. They suggest the presence of a CpG factor and three neighbouring СpH groups comprising cytosines located upstream of rs55798212. At the same time, the CpHs downstream of rs55798212 did not show clear patterns of relations. The factors consisting of CpH sites only moderately correlated with the CpG factor (*r* = −0.01–0.16) and with each other (*r* = −0.08–0.27), suggesting that several different mechanisms influencing methylation might operate within this short segment. Interestingly, the CpH factor with the largest methylation values (chr14:104,060,297–104,060,346) situated at the boundary of a DNase I hypersensitivity site (DHS) designating the end of an active chromatin segment (the UCSC Genome Browser^53^). Subsequent analysis has shown that the CpH grouping could not be explained by ASM.

**Figure 5:**
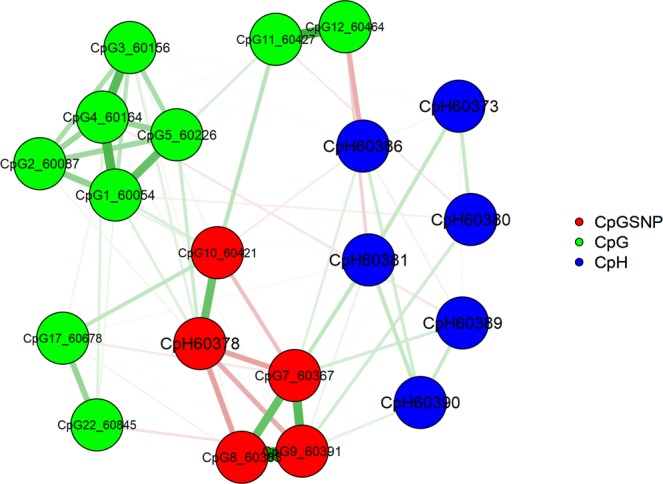
Network of cytosines showing haplotype dependent methylation within Fr_APOPT1_KLC1. The network has been calculated using EBICglasso estimator. Data for the combined sample of patients and controls are presented. The last five digits of a cytosine’s coordinate are shown in the cytosine’s name. Green edges designate positive correlations and red edges designate negative correlations, with the line thickness corresponding to the strength of the connections. Of note, CpH60378 resides at the CpG-SNP rs376515669.

Removing unmethylated CpHs left 61 cytosines for the further analyses. To explore ASM, we used seven polymorphisms (rs67899457* rs55798212* rs376515669* rs151151346* rs147690804* rs12147174* rs143940337) creating three common haplotypes. Average methylation values by group and haplotype and nominally significant *p*-values for each of these cytosines are given in Supplementary Table S7. We observed robust allelic variation in DNA methylation, similar across groups (Table 1), and then conducted an exploratory network analysis of all cytosines with ASM for illustrative purposes. The results indicated that genetically driven methylation at CpG sites mostly co-located with the genetic variants affecting it. In particular, in the case of the schizophrenia risk haplotype TIGACAI, the methylation levels of five CpGs around the mQTL rs67899457 (~200 bp) were intercorrelated and increased compared to the other haplotypes, showing a regional “facilitated” genetic effect^33,54^; while the methylation at each of the consecutively and densely situated CpGs overlapping CpG-SNPs was completely accounted by the absence (rs55798212, rs151151346 and rs12147174) or presence (rs376515669) of the CpG sequence, demonstrating “obligatory” genetic effects. About CpHs, only those situated between these consecutive CpG-SNPs showed ASM, lower methylation taking place in the case of the risk haplotype that destroyed three local CpG sites. In accord with the exploratory factor analysis described above, the network analysis of the sites with ASM revealed several groups of strongly connected consecutive CpGs and no clustering of CpHs (Fig. 5, the associated information can be found in Supplementary Tables S8a–d). This suggests that ASM at these CpH sites might be a mechanistic, non-specific consequence of CpG methylation in the CpG-SNP rich region.

**Table 1:** Methylation levels of cytosines showing haplotype specific methylation in the entire sample after FDR correction.

Cytosine context	Coordinates on chr14	ADAGAGD n = 51 Mean ± s.d.	TIGACAI n = 39 Mean ± s.d.	ADAGAGI n = 41 Mean ± s.d.	Kruskal-Wallis ANOVA, *p*	Mann-Whitney, TIGACAI vs. the others, *p*
CpG	104,060,054	0.53 ± 0.22	0.87 ± 0.17	0.58 ± 0.20	<0.0001	<0.0001
CpG	104,060,087	0.34 ± 0.18	0.65 ± 0.24	0.36 ± 0.21	<0.0001	<0.0001
CpG	104,060,156	0.37 ± 0.21	0.70 ± 0.20	0.41 ± 0.19	<0.0001	<0.0001
CpG	104,060,164	0.42 ± 0.21	0.74 ± 0.19	0.46 ± 0.22	<0.0001	<0.0001
CpG	104,060,226	0.54 ± 0.24	0.86 ± 0.16	0.55 ± 0.21	<0.0001	<0.0001
CpG-SNP	104,060,367	0.88 ± 0.17	0.01 ± 0.02	0.84 ± 0.15	<0.0001	<0.0001
CpH	104,060,373	0.03 ± 0.07	0.01 ± 0.03	0.06 ± 0.11	0.009	
CpH/CpG-SNP	104,060,378	0.04 ± 0.07	0.98 ± 0.07	0.12 ± 0.14	<0.0001	<0.0001
CpH	104,060,380	0.06 ± 0.12	0.00 ± 0.00	0.03 ± 0.06	0.001	0 0.008
CpH	104,060,381	0.08 ± 0.13	0.00 ± 0.00	0.04 ± 0.07	<0.0001	<0.0001
CpG-SNP	104,060,384	0.89 ± 0.18	0.00 ± 0.02	0.89 ± 0.13	<0.0001	<0.0001
CpH-SNP	104,060,386	0.04 ± 0.06	0.01 ± 0.06	0.03 ± 0.08	0.002	0 0.013
CpH	104,060,389	0.05 ± 0.09	0.02 ± 0.08	0.06 ± 0.11	0.015	
CpH	104,060,390	0.05 ± 0.09	0.01 ± 0.05	0.05 ± 0.08	0.010	
CpG-SNP	104,060,391	0.91 ± 0.14	0.04 ± 0.08	0.92 ± 0.12	<0.0001	<0.0001
CpG-SNP	104,060,421	0.06 ± 0.15	0.97 ± 0.07	0.85 ± 0.17	<0.0001	<0.0001
CpG	104,060,427	0.92 ± 0.13	0.99 ± 0.05	0.93 ± 0.11	0.001	0 0.009
CpG	104,060,464	0.95 ± 0.09	0.99 ± 0.04	0.94 ± 0.11	0.007	0 0.011
CpG	104,060,678	0.65 ± 0.20	0.84 ± 0.15	0.76 ± 0.20	<0.0001	<0.0001
CpG	104,060,845	0.87 ± 0.13	0.94 ± 0.10	0.87 ± 0.15	0.005	0 0.002

Notably, the DNA segment harbouring rs67899457 and nearby CpGs is functionally enriched^53^. It resides within DHS and overlaps with several transcription factor binding sites, the list of which can be found in Supplementary Table S9. In contrast, the second segment, harbouring the CpG-SNPs and methylated CpHs, has no such features. These observations are in line with the notion that mQTL lacking CpG-SNPs are enriched in regions of active chromatin and transcription factor binding sites, while mQTL with CpG-SNPs are enriched in quiescent regions and might simply reflect the sequence differences between individuals in heavily methylated regions^30^.

**Figure 6:**
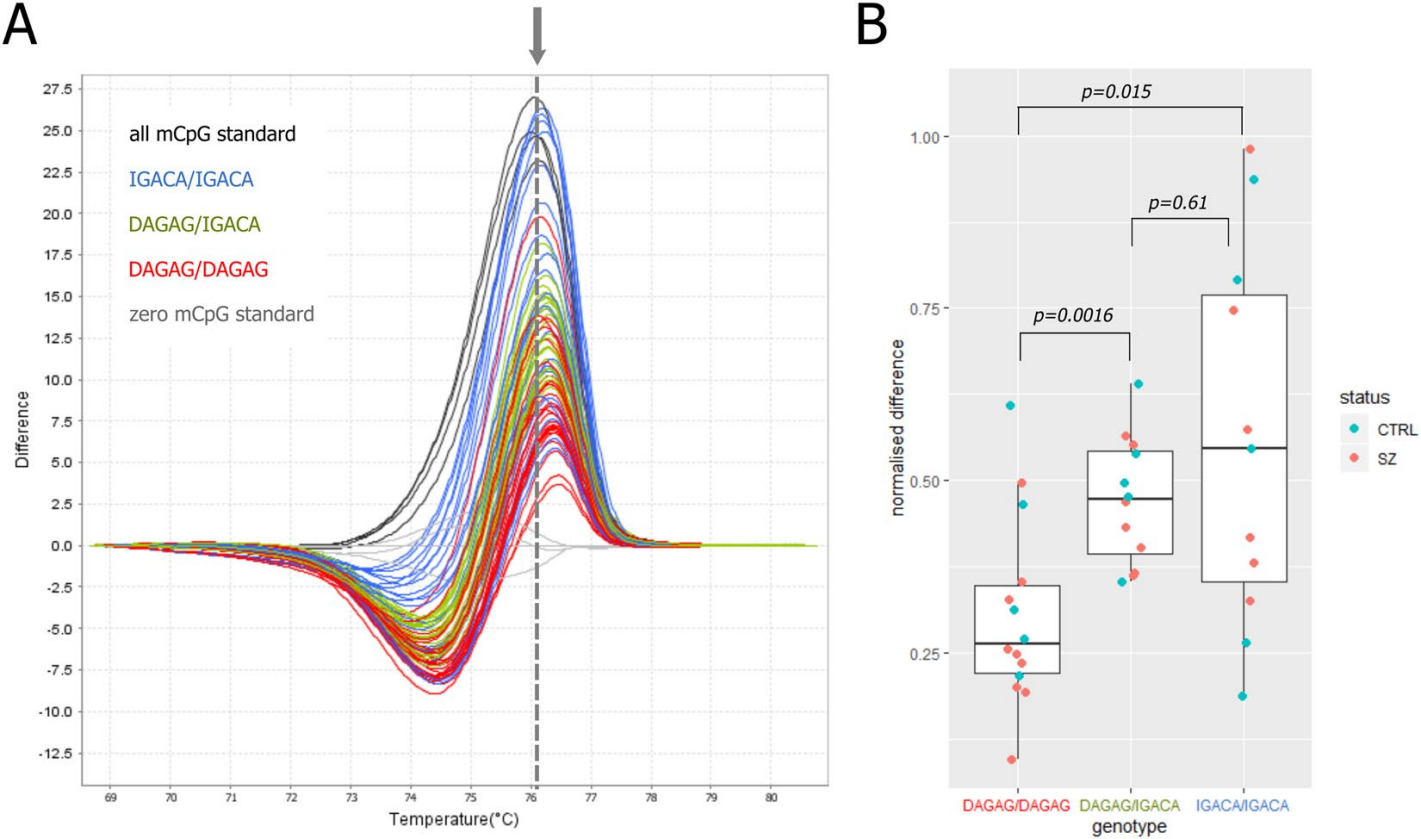
Results of the MS-HRM analysis of allele-specific methylation within Fr_APOPT1_KLC1. (**A**) Melting profiles of the DNA fragment obtained via PCR from bisulfite-converted DNA of 22 schizophrenia (SZ) and 15 control (CTRL) subjects. The fragment includes two CpGs (chr14:104060156 and chr14:104060164). Haplotypes DAGAG and IGACA are formed by polymorphisms rs55798212, rs376515669, rs151151346, rs147690804, and rs12147174. Genotypes are color-coded: IGACA/IGACA in blue, DAGAG/IGACA in green and DAGAG/DAGAG in red. The arrow and the dashed line denote the point of maximum difference between unmethylated and completely methylated standards. (**B**) Boxplot showing distributions of melting difference values (data points on the dashed line in panel A) by genotype. The *p*-values correspond to the two-sided Mann-Whitney U test.

Given the potential functional role of the methylation at the CpGs near rs67899457, we used an independent sample of 37 individuals and methylation-sensitive high-resolution melting (MS-HRM) to validate the local “facilitated” effect of the risk haplotype. To this end, we first genotyped SNPs rs55798212, rs376515669, rs151151346, rs147690804 and rs12147174. In European populations, they form two haplotypes, DAGAG and ICAGA, with ICAGA being part of the above-described schizophrenia risk haplotype TIGACAI. Next, we employed MS-HRM to determine the methylation status for the region chr14:104060091–104060205, containing two CpGs that showed the “facilitated” effect − CpG3_60156 and CpG4_60164. The analysis confirmed that subjects carrying the ICAGA haplotype had significantly higher methylation levels in this region compared to non-carriers (Fig. 6).

Within the Fr_APOPT1_KLC1 fragment, methylation was not associated with diagnosis or cognition after FDR correction (for nominally significant *p*-values see Supplementary Table S7). The haplotype did not influence cognitive functioning either.

### Fr_BAG5

**Figure 7:**
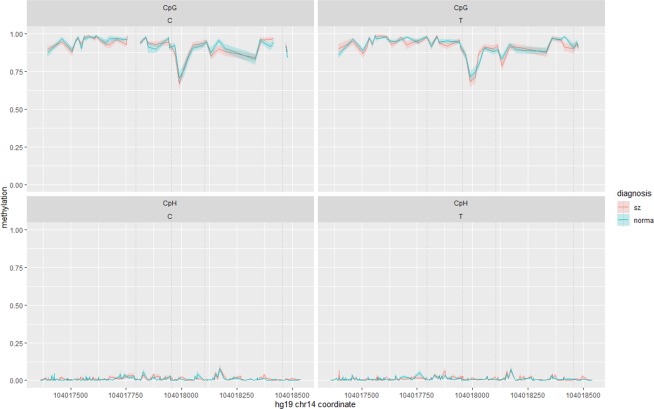
Allele specific methylation at CpG (top panel) and CpH (bottom panel) sites within Fr_BAG5 in schizophrenia patients (red line) and controls (green line). Average methylation with standard errors (coloured shaded areas) is shown separately for the C (the risk allele) and T alleles of rs10141157. Dotted vertical lines show positions of rs12893668, rs10149470, rs10141157 and rs10129426. The empty spaces in the top left panel correspond to the positions of CpG-SNPs rs12893668 and rs10129426, where it is impossible to distinguish unmethylated cytosines from T-alleles.

Methylation levels of 328 cytosines, including 42 CpGs, with the mean coverage of ~17, were determined in 62 patients and 60 controls. Irrespective of age and gender, all CpGs were mostly hypermethylated (M > 50%), while CpHs demonstrated low methylation levels (M < 7.6%) (Fig. 7). We removed unmethylated CpHs and analysed 123 cytosines. Here we were able to determine only one polymorphism, rs10141157, out of nine common local SNPs. However, it is in strong LD with nearby CpG-SNPs rs10149470 (*D*′ = 1.0, *r*^2^ = 1.0), rs10129426 (*D*′ = 1.0, *r*^2^ = 0.90), and rs12893668 (*D*′ = 1.0, *r*^*2*^ = 0.35).

Average methylation values by group and genotype and nominally significant *p*-values for each of these cytosines are given in Supplementary Table S10. In the entire sample, ASM was detected at two CpG-SNPs, rs12893668 and rs10129426. In the patient group, the genotype also influenced a CpG (chr14:104,017,754; *p* = 0.0009) adjacent to the CpG-SNP rs12893668, but the difference between alleles was very small (5%).

After FDR correction, the methylation at individual CpGs and CpHs did not correlate with the diagnosis. In contrast to the other two fragments and in line with the study hypothesis, there were several nominally significant associations between methylation and cognition, however none of them survived correction for multiple testing. Genotype did not influence cognition (for nominally significant *p*-values see Supplementary Table S10).

## Discussion

In this work, we evaluated the level of methylation of each cytosine, in both the CpG and non-CpG context, within three fragments of one of the top schizophrenia risk loci on chromosome 14 overlapping with an intelligence GWAS locus. Each fragment was chosen based on its potential regulatory role in the expression of nearby genes, primarily *BAG5*, *APOPT1* and *KLC1*, and the enrichment for CpG-SNPs. The latter is important due to the CpG-SNP frequent roles as mQTL and eQTL and also because methylation at CpG-SNP sites is highly correlated in the blood and brain, which partially removes the limitations of using blood as a surrogate for brain in research of psychiatric diseases^13,32^. We applied the bisulfite conversion-based method (SMRT-BS) to assess cytosine methylation in peripheral blood of controls and patients and obtained average values of methylation, which were in good agreement with those reported for CpGs in neutrophils and monocytes in the publicly available WGBS datasets^55^ (Supplementary Fig. S1). Using PacBio’s long reads enabled us to directly access allelic information, which in turn allowed for testing differences in methylation between patients and controls considering their haplotypes.

Previously, the array based MWASs of blood and brain tissues have detected differential methylation of several CpGs within this locus in schizophrenia^8,10^. The identified differentially methylated positions are outside the fragments investigated in the present study. For instance, hypomethylation of cg13247935 (chr14:104,116,713) located in *KLC1* has been found in the brain of patients, with a difference in methylation between cases and controls of 2.47%^10^. At the same time, none of seven CpGs tested by the MWASs in the common promoter of *KLC1, BAG5* and *APOPT1* has demonstrated differential methylation. In addition, Hannon *et al*.^8^ have not found evidence of a common causal genetic variant for schizophrenia and methylation of these seven CpGs in peripheral blood. Our results extend the MWAS data to the entire promoter, showing very low level of methylation of all CpG and CpH sites regardless of the presence of the schizophrenia risk haplotype. The pattern of methylation that we have observed in the second fragment, located in the intergenic area downstream of *BAG5*, is also very consistent across groups and genotypes, with hypermethylated CpGs and hypomethylated CpHs. Genetic effects on methylation have been found only at two CpG-SNP sites, where the risk allele associates with the absence of the CpG sequence. Of note, this region is now identified as an enhancer regulating the *CKB* gene expression (GeneHancer ID: GH14J103549)^46^.

In contrast, the fragment in the *KLC1* fourth intron, within the enhancer regulating the common promoter of *BAG5*, *APOPT1* and *KLC1*, has shown robust ASM of many CpGs and some CpHs. In that, the schizophrenia risk haplotype has differed from the other two haplotypes by the increased methylation of several CpGs, including those surrounding the mQTL rs67899457, and by the lack of methylation at several consecutive cytosines residing at CpG-SNPs and CpHs. While the lack of methylation seems simply to reflect DNA sequence characteristics, especially given no known functional significance of this segment, the methylation around rs67899457 is of interest due to its location in DHS. Moreover, rs67899457 is an eQTL in blood and brain tissues (information about eQTL was retrieved from the NESDA NTR Conditional eQTL Catalog^47^ and GTEx(v8)^48^ and summarized in Supplementary Table S1). Interestingly, its T-variant, which is part of the schizophrenia risk haplotype, is associated with a higher expression of several genes in the blood and a lower expression of *APOPT1* in the brain^48^.

Within this enhancer fragment in *KLC1*, we observed ASM but no difference between patients and controls in methylation. These results can be considered under two different scenarios of the methylation role in the disease risk. First, they do not support the hypothesis of the genetically independent role of the local methylation in schizophrenia, as patients and controls with the same haplotypes have shown similar levels of methylation. Second, the results do provide evidence for ASM in this fragment. The presence of ASM suggests that in a sufficiently large sample there might be a difference in methylation between cases and controls owing to the accumulation of the schizophrenia risk haplotypes in patients. And this would be in accordance with the proposed role of ASM as a marker or a mediator of the genetic effects on disease susceptibility. Given this, further investigation of the role of methylation at CpG sites surrounding rs67899457 in schizophrenia is warranted.

There are some limitations to note. Given tissue and cell-type specificity of methylation, two methodological concerns regarding the use of blood in methylation studies of mental traits are widely debated in the literature. These are the use of blood as a surrogate for brain and the complex cellular composition of the blood^56,57^. As mentioned earlier, testing methylation under genetic control (mQTL), which is a focus of our study, improves the interpretability of findings but does not remove the limitations completely. Further, about the credibility of the results, we used the SMRT-BS method, which is highly reproducible and concordant with other quantitative CpG methylation methods^34,35^. In addition, we validated the haplotype-dependent ASM near rs67899457 using MS-HRM and an independent sample. Moreover, our data on methylation within each fragment are in agreement with those obtained previously using large samples and other approaches to assessing DNA methylation^8,30,55^. Even so, replication of both the positive and negative results using independent samples and another sequencing method enabling the analysis of long amplicons is desirable. Our sample size, albeit common for studies based on next-generation sequencing, could have been too small to capture some relevant effects. Of note, however, the desirable sample size is unknown because there is no information as to what a biologically significant difference in methylation levels is. Then, when considering the results, one should keep in mind that CpH methylation is strand-specific, showing greater asymmetry in introns and highly transcribed genes^15^. Finally, the schizophrenia risk locus selected for the study spans ~200 kb. Constrained by the high cost of the methodology used, we analysed only three fragments, about 1 kb each, and their choice was to some extent arbitrary. Other fragments with marks of active chromatin deserve attention in future studies.

In summary, sequencing-based profiling of haplotype dependent DNA methylation in three regions within the genomic locus ranked 13th in the schizophrenia PGC2 GWAS^1^ has revealed neither CpG nor CpH sites with altered methylation in schizophrenia. Likewise, no effects of local haplotypes and methylation on cognitive deficits have been found. At the same time, we have shown that the schizophrenia risk haplotype within the fourth intron of *KLC1* associates with specific methylation profile irrespective of the diagnosis and confirmed ASM for cytosines located in the vicinity of rs67899457. These genetically associated DNA methylation variations may be related to the pathophysiological mechanism differentiating the risk and non-risk haplotypes and merit further investigation.

## Methods

### Sample

The present study is part of a larger research on epigenetics of cognitive deficits in schizophrenia. Participants were selected from a database of the Mental Health Research Center (MHRC) in Moscow, as previously described^58^. In brief, cases were recruited from inpatient units of local psychiatric clinics. Healthy controls were recruited by word of mouth without any participation fee, mainly from employees of research institutes, hospitals and students. Candidates were included if they were Caucasian, aged 18–45 years, completed at least a secondary school (11 years), and had no history of brain injury, psychoactive medication or other medical conditions that may affect cognitive functions.

The sample consisted of 77 patients with schizophrenia (mean age, s.d., 26.87 ± 6.72 years, 49% women) and 70 healthy subjects without a family history of psychosis (mean age, s.d., 27.14 ± 6.92 years, 51% women). Sixty-seven patients suffered from schizophrenia (F20 according to the International Classification of Diseases, the 10th revision), the others were diagnosed with schizophrenia-spectrum disorders F21 (n = 2), F23 (n = 3) and F25 (n = 5). All patients received complex antipsychotic medication regimes. The mean disease duration was 5.76 ± 6.01 years. Sixty-two percent of patients and 90% of controls were university students or already had a university degree. According to self-reports, 34% of patients and 29% of controls were current or former smokers.

All subjects donated blood samples for DNA extraction, provided personal demographic data and performed six cognitive tests assessing processing speed, verbal memory, and executive functions. A composite cognitive index, which was a T-score averaged across the tests, was calculated for each participant. The assessment procedure is described in detail in ref. ^59^; the tests are listed in Supplementary Table S3.

All subjects gave written informed consent to participate in the research. The study was conducted in accordance with the principles of the Declaration of Helsinki and approved by the MHRC Ethics Committee.

### SMRT-BS methylation analysis

DNA was extracted from 1 ml of whole blood. The entire procedure of the DNA methylation analysis is described in detail in Kondratyev *et al*.^60^. DNA was extracted with the DNeasy Blood and Tissue Kit (Qiagen, USA) and bisulfite converted with the EpiGentek Methylamp DNA Modification Kit (EpiGentek, USA) according to the manufacturers’ instructions. The primers for each fragment were designed with the primer3 software. Their sequences are shown in Supplementary Table S1. PCR with bisulfite-converted DNA was performed according to the modified (“panhandle”) SMRT-BS method^34,60^. The modified method employs the suppressive hybridization (“panhandle”) PCR approach to enhance the specificity of the reaction and “Y-adapter” ligation strategy for barcoding. PCR was performed using 20 ng of the converted DNA, 5 nmol of each primer,1 μmol of 5′-phosphorylated primer pU1 5′-P-GCAGTCGAACATGTAGCTGACTCAGGTCAC, 200 nmol of dNTP, 1 mg/mL bovine serum albumin, and 2.5 units of HotTaq-polymerase (Sileks, Russia) in a total volume of 12.5 μL. PCR included the following steps: (1) initial denaturation, 94 °С, 10 min; (2) specific PCR, 5 cycles: 94 °С, 20 s; 55 °С, 1 min; 64 °С, 4 min; (3) “panhandle” PCR, 37 cycles: 94 °С, 20 s; 64 °С, 2 min; (4) 64 °С, 10 min. To create unique barcodes, we employed 96 unique combinations of eight sequences of the type 5′-phosphate – CGAGTAGTGT - TC- unique five-letter barcode-CAAGGCACACAGGGGATAGG and 12 sequences of the type 5′-CATCTCATCCCTGCGTGTC - unique five-letter barcode -CTACACTACTCG-T. The CCS library preparation (ligation of “SMRTBell” adapters with SMRTbell Template Prep Kit, PacBio, USA) and sequencing were carried out by the University of Washington PacBio Sequencing Services with PacBio RSII (P6/C4 chemistry) and followed by post-sequencing data preparation and quality control. Adapter trimming and barcode demultiplexing were performed with the cutadapt programme. Only reads with a quality score of no less than Q30 were utilised in subsequent analysis. No errors in barcode sequences were allowed during demultiplexing. The bismark and bowtie2 software was applied for the alignment of the filtered reads to the reference human genome (hg19) and to determine the rate of methylation of individual CpGs/CpHs per haplotype. Filtration of under-converted DNA (threshold of unconverted CpH <5%) and deduplication were performed with the perl script. The quality control resulted in 2192 reads for Fr_BAG5_APOPT1, 2245 reads for Fr_APOPT1_KLC1, and 2397 reads for Fr_BAG5.

For each subject, the methylation levels (M) of individual cytosines were calculated as the ratio of reads with unconverted cytosine to the total amount of reads for a given cytosine. Haplotypes were determined directly from sequencing data. Only samples with the minimum 5× read depth per haplotype were analysed.

### MS-HRM validation of the haplotype effect in Fr_APOPT1_KLC1

An independent sample comprising 22 schizophrenia patients and 15 controls were derived from the MHRC database using the above-described criteria. DNA was extracted from whole blood.

Primers for genotyping by HRM were designed to capture the region chr14:104060342–104060475 containing SNPs rs55798212, rs376515669, rs151151346, rs147690804 and rs12147174, which form two haplotypes in European populations: DAGAG and ICAGA. The sequences of the primers were as follows: “DAGAG1_for” CAGGCTGGTCTCAAACTCCT and “DAGAG1_rev” GTGCAGCTTCCGGTACATTA. The polymerase reaction was set in 20 ul with 25 ng DNA, 200 nM primers, 20× EvaGreen dye (Biotium, USA), 200 uM dNTP and 0.5 units of the HotStart Taq polymerase in 10x Taq Turbo buffer (Evrogen, Russia). The PCR programme included (1) preheating at 95 °C for 3 minutes; (2) 40 cycles of 95 °C for 10 seconds; 62 °C for 10 seconds; 72 °C for 20 seconds. Melting curves were collected by slowly (0.025 °C/s) increasing the reaction temperature from 60 °C to 95 °C. The PCR was performed in the Applied Biosystems QuantStudio 7 instrument. The positive controls for the HRM genotyping were obtained through imputation (IMPUTE2) with 1000 genome reference panel (EUR) in the laboratory-owned chip-genotyped sample. Of note, while the DAGAG1_for primer has multiple annealing sites across the genome, the PCR product of the pair of primers used is specific to the region of interest under the described reaction conditions.

For MS-HRM, primers were designed to the region chr14:104060091–104060205 of bisulfite-converted DNA (Methylamp kit, EpiGentek, USA). The sequences of the primers were as follows: “876_115_for” GGGTTATTTTAGGTTAGTGAAAATAGGA and “876_115_rev” CAAAAATCATACCACTACACTCCAAC. The region includes two CpGs: CpG3_60156 and CpG4_60164 (see Supplementary Table S7). The PCR were set in duplicates. Unmethylated and completely methylated standards from EpiTect PCR Control Set (Qiagen, USA) were used. The polymerase reaction was set in 20 ul with 10 ng of bisulfite-converted DNA, 200 nM primers, 20x EvaGreen dye (Biotium, USA), 200 uM dNTP and 0.25 units of the EpiMark Hot Start polymerase (NEB, USA). The reaction programme included preheating at 95 °C for 3 minutes; 40 cycles of 95 °C for 15 seconds, 61 °C for 20 seconds, 65 °C for 40 seconds; HRM programme: 60 °C–95 °C, 0.025 °C/sec. The PCR was performed in the Applied Biosystems QuantStudio 7 instrument. The raw melting data were analysed with the built-in QuantStudio Realtime Software v1.1. The difference data were used to assess ASM.

### Statistical analysis

We analysed each fragment as follows. First, we searched for ASM in the entire group and then separately in patients and controls using the Kruskal-Wallis tests. The Mann-Whitney U test was applied for post-hoc comparisons of haplotypes and to test sex differences in methylation. Spearman correlations between age and methylation were also examined. Next, to determine the association of methylation with case-control status, logistic regression was used, with a separate model adjusted for sex, age and haplotype was built for each methylated cytosine (1% < M < 99%). Since the coverage varied, which might affect the detection of a small difference between groups^61^, we additionally compared subsamples of patients and controls using the Wilcoxon matched pairs test. For this analysis, each patient was associated with a control of the same haplotype and about the same coverage. Of note, data on smoking were not available for all subjects. For this reason, the effects of smoking at every site had been preliminary evaluated to assess whether it could represent a confounder. No significant effects were found, and we therefore did not consider smoking in subsequent analysis.

The effects of haplotypes and methylation on the cognitive index were evaluated by general linear models with the inclusion of age, gender and diagnosis as covariates. The influence of haplotypes was tested in two ways. First, a genotype factor included all combinations of haplotypes in the sample. Next, the genotype factor was defined as the number of alleles with a “risk haplotype”. For each fragment, the latter comprised variants correlating with the schizophrenia risk allele T at rs12887734 (Supplementary Table S2). A separate model was built for every cytosine, with methylation values being averaged over two alleles in heterozygotes.

Additional analyses were performed as follows. The exploratory factor analysis included the parallel analysis to evaluate the number of factors to retain and the oblique (Promax) rotation. The network analysis used EBICglasso estimator, with applying the nonparanormal transformation to make all data normally distributed.

For each type of analyses, two-tailed tests were used, and the associations were considered significant if they passed a false discovery rate (FDR) threshold of 0.05. Most analyses were carried out with JASP 0.11.1.0^62^.

## Data Availability

All data generated during the present study are available from the corresponding author on reasonable request.

## Supplementary.Information

Supplementary Information.
